# Supplementary material for: Issues and Opportunities Associated with Trophy Hunting and Tourism in Khunjerab National Park, Northern Pakistan
Source: Animals (Basel). 2020 Apr 1;10(4):597. doi: 10.3390/ani10040597 (PMC7222849; doi:10.3390/ani10040597)
Supplement: Supplementary file 1 [file animals-10-00597-s001.pdf]

Table S1. Questions for focus group included in the current trophy hunting and mass tourism FDGs questionnaire.

S.No	Question	Response format
1.	How you perceive predators presence in the trophy hunting area?	BA = Beneficial Animals PA= Pest Animals, DK = Don't Know OS= Others Specify
2.	How many among you would like to see change in the population of snow leopard species in future?	Increase = Decrease= Eliminate= No change =
3.	How many among you would like to see change in the population of Wolves species in future?	Increase = Decrease= Eliminate= No change =
4.	How many among you would like to see change in the population of Ibex species in future?	Increase = Decrease= Eliminate= No change =
5.	How many among you would like to see change in the population of blue sheep species in future?	Increase = Decrease= Eliminate= No change =
6.	Can you narrate stories of snow leopards killed in the last 8 years in this valley?	Open
7.	Compensation scheme available: for loss due to wild animals?	Yes /no
8.	Suggested measures for the snow leopard conservation (Key suggestions: to make a balance)	Open
9.	What human–snow leopard conflict mitigation measure you propose for this area	Open
10.	What do you think about trophy hunting in this valley in last 26 years?	Open
11.	Where the money from trophy hunting is used?	R= Roads I= Infrastructure E= Education L= Livelihood O= Others, Please specify
12.	Are there any drawbacks or disadvantages of trophy hunting to wildlife in the study area?	Open
13.	Information about tourists and vehicles entering into national park since 1999?	Open

14.	Do you use any funds for awareness of tourists?	Yes /no
15.	Had you observed any wild animals killed due to cars /vehicles?	Open
16.	What are the problems related to tourism inside the park?	Open

Table S2: Details for Trophy hunting of Ibex and economic contribution in KVO area.

S.No	Year	No. of ibex hunted	Total amount in US $	1 US $= PKR (Conversion Rate)	Community share		Government share	
					@ 75 %	@ 80 %	@ 25 %	@ 20%
1	1993–1994	1	497	30.2	372.75	—	124.25	—
2	1994–1995	1	485	30.9	363.75	—	121.25	—
3	1995–1996	1	446	33.6	334.5	—	111.5	—
4	1996–1997	1	385	38.9	288.75	—	96.25	—
5	1997–1998	1	347	43.2	260.25	—	86.75	—
6	1998–1999	2	2520	46.8	1890	—	630	—
7	1999–2000	1	300	50.0	225	—	75	—
8	2000–2001	4	3057	58.4	—	2445	—	611
9	2001–2002	6	1954	61.4	—	1563	—	390.8
10	2002–2003	5	2137	58.5	—	1709.6	—	427.4
11	2003–2004	0	0	0	0	0	0	0
12	2004–2005	9	6946	59.4	—	5557	—	1389
13	2005–2006	11	15008	59.9	—	12007	—	3001.6
14	2006–2007	10	17038	60.6	—	13630	—	3408
15	2007–2008	12	36500	62.5	—	29200	—	7300
16	2008–2009	12	22158	78.5	—	17726	—	4432

17	2009–2010	6	16134	83.8	—	12908	—	3226
18	2010–2011	5	13678	85.5	—	10943	—	2735
19	2011–2012	3	8700	89.2	—	6960	—	1740
20	2012–2013	2	5400	95.4	—	4320	—	1080
21	2013–2014	16	32478	101.6	—	25982	—	6496
22	2014–2015	18	29975	101	—	23980	—	5995
23	2015–2016	0	0	102.7	0	0	0	0
24	2016–2017	29	67510	104.7	—	54008	—	13502
25	2017–2018	14	43310	105.4	—	34648	—	8662
**Grand Total**		**170**	**326963**			**261321**		**65641**

Table S3: Details of Blue sheep trophy hunting and economic contribution in KVO area.

S.No	Year	Total No. of Trophy Hunted.	Pakistani hunters	Foreign hunters	Total amount in US $	Community Share @ 80 in US $	Government Share @ 20 in US $
**1**	2004–2005	2	1	1	10000	8000	2000
**2**	2005–2006	1	0	1	5000	4000	1000
**3**	2006–2007	0	0	0	0	0	0
**4**	2007–2008	2	0	2	30900	24720	6180
**5**	2008–2009	0	0	0	0	0	0
**6**	2009–2010	0	0	0	0	0	0
**7**	2010–2011	0	0	0	0	0	0
**8**	2011–2012	0	0	0	0	0	0
**9**	2012–2013	3	0	3	20100	16080	4020

**10**	2013–2014	1	0	1	8000	6400	1600
**11**	2014–2015	2	1	1	13600	10880	2720
**12**	2015–2016	0	0	0	0	0	0
**13**	2016–2017	1	0	1	8500	6800	1700
**14**	2017–2018	0	0	0	0	0	0
	**Total**	**12**	**2**	**10**	**96100**	**76880**	**19220**

Supplementary Table S4: Number of tourists and income from the entry fees into Khunjerab National Park.

S.No	Year	Total No. of visitors	Income in US $	Percentage of the total	1 US $ = PKR conversion rate	Income in PKR
**1**	1999	1382	1377	0.4 %	50.0	68860
**2**	2000	35148	30609	9 %	58.4	1787624
**3**	2001	68178	38638	11 %	61.4	2372373
**4**	2002	13331	8160	2 %	58.5	477360
**5**	2003	11362	8926	3 %	57.6	514183
**6**	2004	15651	17014	5 %	59.4	1010666
**7**	2005	14102	19488	5 %	59.9	1167326
**8**	2006	17059	20826	6 %	60.6	1262075
**9**	2007	14900	16830	5 %	62.5	1051890
**10**	2008	11922	12128	3 %	78.5	952064
**11**	2009	10342	8181	2 %	83.8	685622
**12**	2010	4644	4022	1 %	85.5	343908
**13**	2011	7930	12232	3 %	89.2	1091100
**14**	2012	7747	11223	3 %	95.4	1070654

**15**	2013	10145	11732	3 %	101.6	1192003
**16**	2014	15037	13852	4 %	101	1399112
**17**	2015	41085	23825	7 %	102.7	2446878
**18**	2016	93421	44831	13 %	104.7	4693852
**19**	2017	139056	108307	30 %	105.4	11415572
**20**	2018	145633	101895		121.7	12400620
**Total**		**678,075**	**457,137**	**100 %**	**——**	**474,037,42**
